# Factors associated with poor sleep quality among construction workers in Arba Minch town, Ethiopia: A cross‐sectional study

**DOI:** 10.1002/hsr2.1715

**Published:** 2023-11-21

**Authors:** Debisa E. Wendimu, Solomon G. Meshesha

**Affiliations:** ^1^ Malaria and Neglected Tropical Disease Directorate Armauer Hansen Research Institute (AHRI) Addis Ababa Ethiopia; ^2^ Clinical Trial Directorate Armauer Hansen Research Institute (AHRI) Addis Ababa Ethiopia

**Keywords:** construction workers, occupational health and safety, Pittsburgh Sleep Quality Index, sleep quality

## Abstract

**Background and Aims:**

Sleep is critical for human physical and cognitive health. Even though poor sleep quality had a major negative impact on workers' health and safety, there is a scarcity of study that attempts to demonstrate its magnitude and causative factors in labor‐intensive environments.

**Methods:**

A cross‐sectional study design was employed. A total of 423 individuals were selected using a simple random sampling technique, starting from April 1, 2020 to May 1, 2020. Interviews were administered using the validated and pretested Pittsburgh Sleep Quality Index (PSQI) tool. EpiData version 4.4.3.1 was used for data entry and SPSS version 25 for analysis. To associate factors with poor sleep quality binary logistic regression model was fitted at 95% confidence interval (CI). A *p* < 0.25 and *p* < 0.05 were used to declare association in bivariable and multivariable analysis, respectively.

**Results:**

A total of 415 building construction workers take part in this study, with a response rate of 98.1%. A PSQI global score showed 66.3% (95% CI: [0.63, 0.71]) of construction workers suffering from poor sleep quality. Working hours, work experience, previous injury status, managerial support on occupational safety and health, cigarette smoking, and job satisfaction were the factors that were associated with poor sleep quality significantly.

**Conclusions:**

Two‐thirds of construction workers suffer from poor sleep quality. Risk factors include shorter working years, longer working hours, prior injury, lack of managerial support, cigarette smoking, and low job satisfaction. Efficient health education and promotion through taking the identified associated factors into account could help reduce poor sleep quality.

## 1 BACKGROUND

Sleep is a basic behavior found throughout the animal kingdom, and it is essential for maintaining physical health and cognitive function in humans.^1^ The ideal amount of sleep for adults to function well, prevent sleep debt, and avoid daytime drowsiness is around 7–8 h and at least 10 h for school‐aged children and adolescents.^2^ One of the most essential things our body need is good sleep quality.^3^ Workers with poor sleep quality experience a variety of health issues, and even premature mortalities.^4^, ^5^, ^6^

According to large‐scale global research on sleep disorders conducted in eight Asian and African countries, 17% of the population in these developing countries suffers from sleep problems.^7^ In Tanzania, Ghana, and Kenya, overall rates of poor sleep quality varied from 8.3% to 12.7%, making it more common than several chronic disorders and infectious diseases.^7^ In Ethiopia, 53% of the population was estimated to have poor sleep quality.^8^

Poor sleep quality is a serious issue that construction workers face regularly. According to studies conducted in Korea and China, around 63% and 30%, respectively, of construction workers struggle with poor sleep quality.^9^, ^10^ As a result of sleep disorders, they were more likely to experience work‐related injuries and fatalities.^11^, ^12^, ^13^ Very sleepy workers are 70% more likely to be engaged in workplace accidents than their nonsleep‐deprived coworkers.^14^ An increased risk of job injuries can also result from long workdays and inadequate sleep. Besides workers' health, poor sleep quality has far‐reaching economic consequences on the nation's economy. Globally, lack of sleep and erratic sleeping patterns caused billions of dollar loss.^15^

Regardless of the fact that poor sleep quality had a major negative impact on workers' health and safety, few research have attempted to demonstrate its magnitude and causative factors in labor‐intensive environments. This lack of information makes it difficult to establish specific interventions and care programs to safeguard construction workers' overall health and safety. Therefore, this study aimed to close the gap of information regarding poor sleep quality prevalence among construction workers and the factors associated with it.

## 2 MATERIALS AND METHODS

### 2.1 Study design and setting

An institution‐based cross‐sectional study design was employed, and data were collected starting from April 1, 2020 to May 1, 2020. This study was conducted in Arba Minch town, the capital of Gamo zone, Southern Nation's Nationalities and Peoples Regional State of Ethiopia. It has a total population of 113,297 people. It lies 250 and 435 km from the regional city of Hawasa and the capital city of Ethiopia, Addis Ababa, respectively. According to information obtained from Gamo zone urban development and construction office, at the time of data collection, there were five buildings which have been under‐construction by construction firms licensed with 1–8 grades.

### 2.2 Population and eligibility criteria

All construction workers in Arba Minch town were the source population. Selected construction workers who were involved in the construction process for the past 12 months were the study population.

### 2.3 Sample size determination

The sample size was calculated using Epi Info version 7 and the single population proportion formula. A 95% confidence level, 5% marginal error, and 50% poor sleep quality proportion were assumed, as no previous studies had been conducted in this setting. A nonresponse rate of 10% was also considered, resulting in a sample size of 423.

### 2.4 Sampling procedure

Of the 488 eligible construction workers at five construction sites, which were under construction by Grade 1–8 licensed construction firms in the study area, 423 were selected using a simple random sampling technique with a lottery method. The sample size was proportionally allocated to the five construction sites based on the number of workers at each site.

### 2.5 Study variables

Sleep quality status is our outcome variable. Whereas sociodemographic variables (age, sex, marital status, educational status, religion, and monthly income), work‐related variables (employment status, type of work, work experience, work hour/day, previous injury status, and managerial support on occupational safety and health [OSH]), and behavioral related variables (khat chewing, alcohol drinking, cigarette smoking, knowledge of OSH, attitude on OSH, job satisfaction) are our response variables.

### 2.6 Operational definition

#### 2.6.1 Sleep quality

Pittsburgh Sleep Quality Index (PSQI) was used to measure sleep quality. This tool has seven components, and each of the seven components is awarded a score ranging from 0 to 3 (0: *being no problem*). The global PSQI scores vary from 0 to 21. Finally, those with a global PSQI score of greater than 5 were deemed to have poor sleep quality.^16^

### 2.7 Data collection tools and procedure

The tool for data collection was adopted from multiple works of literature,^16^, ^17^, ^18^ and the questionnaire was administered by the interviewer. To collect the status of the outcome variable a validated tool (PSQI) was utilized.^18^ Three BSc nurses carried out the data collection procedure, and one BSc nurse professional supervised the procedure. The collected data were reported to the supervisor every 2 days. The completed questionnaires were sent to the principal investigator after the supervisor rechecked the filled questionnaires.

### 2.8 Data quality control

Data collectors and supervisors were trained on data collection processes, methodologies, and ethical considerations. The questionnaire was pretested on 5% of the study's sample size among construction site employees in Mirab‐Abaya, Southern Ethiopia. The pretest ascertained the consistency of the research tool. All the questionnaires were verified daily to see whether they were accurately filled or not.

### 2.9 Data management and statistical analysis

Before analyzing the raw data with SPSS statistical software version 25.0, EpiData version 4.4.3.1 was used to code and enter the data into the computer. Then, it was cleaned and checked by running frequency. The data were described using frequency, proportion, mean, and tables.

Multicollinearity (variance inflation factor) and model fitness (Hosmer and Lemeshow) were checked before applying and interpreting the binary logistic regression model. In bivariable analysis, independent variables with a *p* < 0.25 were included in multivariable analysis, and variables were recognized as statistically significant if their *p* < 0.05.

### 2.10 Ethical consideration

The Institutional Review Board of Arba Minch University, College of Medicine and Health Sciences, provided ethical clearance. Every research participant was given written informed consent after the study collector provide them a brief description of the importance of the study (Supporting Information S1: Appendix 1). For individuals under the age of 18, written informed consent was obtained from their parents. The participants' confidentiality was maintained throughout the whole investigation.

## 3 RESULTS

### 3.1 Sociodemographic characteristics

From 423 construction workers, 415 responded with complete data making the response rate 98.1%. The majority (65.8%) of the respondents were males. The participants' mean (SD) age was 25.80 (±6.07) years, with the majority (47.7%) falling between the ages of 25 and 49. Most of the workers (40.2%) had only a primary school education (Table 1).

**Table 1:** Sociodemographic characteristics of building construction workers in Arba Minch town, Southern Ethiopia, 2020.

Variables	Frequency (*n*)	Percent (%)
Sex		
Male	273	65.8
Female	142	34.2
Age (years)		
≤19	56	13.5
20–29	260	62.7
30–39	87	21.0
≥40	12	2.9
Marital status		
Single	254	61.2
Married	147	35.4
Divorced/widowed/separated	14	3.4
Religion		
Orthodox	183	44.1
Protestant	150	36.1
Muslim	31	7.5
Catholic	43	10.4
Others^a^	8	1.9
Educational status		
No formal education	2	0.5
Primary school	167	40.2
Secondary school	153	36.9
Diploma and above	93	22.4
Average monthly income (Birr)		
1000–1999	73	17.6
2000–3999	309	74.5
Above 3999	33	8.0

^a^ Jehovah's witness and individuals who have no religion.

### 3.2 Work‐related characteristics

Of the total respondents, 59.3% of the workers were temporarily employed. Three‐quarters of those interviewed had ≤2 years of work experience. Fifty‐eight percent of the participants had poor managerial support on OSH. Sixty percent of the study participants had worked for more than 8 h/day (Table 2).

**Table 2:** Work‐related characteristics of building construction workers of Arba Minch town, Southern Ethiopia, 2020.

Variables	Frequency (*n* = 415)	Percent (%)
Employment status		
Temporary	246	59.3
Permanent	20	4.8
Micro and small‐scale enterprises (contract)	149	35.9
Type of work		
Daily laborer	207	49.9
Plasterer	42	10.1
Carpenter	65	15.7
Mason	65	15.7
Others^a^	36	8.7
Experience (years)		
≤2	318	76.6
>2	97	23.4
Work hours/day (h/day)		
≤8	166	40.0
>8	249	60.0
Previous injury status		
Yes	178	42.9
No	237	57.1
Managerial support on OSH		
Good	172	41.4
Poor	243	58.6

Abbreviation: OSH, occupational safety and health.

^a^ Driver/operator, site engineer, painter, and welder/electrician.

### 3.3 Behavioral‐related characteristics

Among the study participants, 28.2%, 11.8%, and 8.0% of the workers were khat chewers, alcohol drinkers, and cigarette smokers, respectively. Most of the respondents (69.2%) have a positive attitude toward OSH (Table 3).

**Table 3:** Behavioral characteristics of building construction workers of Arba Minch town, Southern Ethiopia, 2020.

Variables	Frequency (*n* = 415)	Percent (%)
Khat chewer		
Yes	117	28.2
No	298	71.8
Alcohol drinker		
Yes	49	11.8
No	366	88.2
Cigarette smoker		
Yes	33	8.0
No	382	92.0
Knowledge of OSH		
Good knowledge	162	39.0
Medium level knowledge	100	24.1
Poor knowledge	153	36.9
Attitude on OSH		
Positive attitude	287	69.2
Negative attitude	128	30.8
Job satisfaction		
Good	195	47.0
Poor	220	53.0

Abbreviation: OSH, occupational safety and health.

### 3.4 Prevalence of sleep quality

The PSQI global score (>5) showed that 66.3% of construction workers suffer from poor sleep quality in this study. The respondent went to sleep at 9:57 p.m. on average and awoke at 6:46 a.m. The participants' mean (SD) night sleep duration was 7.86 h (±0.94), and only 17 (4.1%) of the individuals subjectively claimed to have very bad sleep quality. The average sleep latency of the participants was 43 min (SD ± 23.48). More than one‐third (34.7%) of the study participants reported that they had ≤7 h duration of sleep per night. In this study, 15 (3.7%) of the participants had a low habitual sleep efficiency (<65%), and 19 (4.5%) of them used sleep medication less than once per week. The mean (SD) values for sleep disturbances and daytime dysfunction were 1.11 (±0.39), and 1.39 (±1.12), respectively (Table 4).

**Table 4:** Sleep quality components scores among building construction workers of Arba Minch town, Southern Ethiopia, 2020.

Variables	Frequency (*n* = 415)	Percent (%)
Sleep duration (h)		
>7	271	65.3
6–7	80	19.3
5–6	43	10.4
<5	21	5.0
Sleep latency		
0	15	3.6
1	208	50.1
2	143	34.5
3	49	11.8
Daytime dysfunction		
0	130	31.3
1	88	21.2
2	141	34.0
3	56	13.5
Habitual sleep efficiency (%)		
>85	278	67.1
75–84	93	22.3
65–75	29	6.9
<65	15	3.7
Subjective sleep quality		
Very good	140	33.8
Fairly good	161	38.7
Fairly bad	97	23.4
Very bad	17	4.1
Sleep disturbance		
0	10	2.3
1	324	78.1
2	56	13.5
3	25	6.0
Use of sleep medication		
Not during the past month	392	94.5
Less than once a week	19	4.5
Once or twice a week	4	1.0
Three or more times a week	0	0.0
Sleep quality score		
Good sleep	140	33.7
Poor sleep	275	66.3

### 3.5 Associated factors

In bivariable analysis, poor sleep quality was associated (*p* < 0.25) with the age of the respondent, marital status, monthly income, employment status, type of work, work experience, work hour/day, previous injury status, managerial support on OSH, khat chewing, alcohol drinking, cigarette smoking, knowledge of OSH, attitude on OSH, and Job satisfaction. All these variables were added to the multivariable analysis. In the multivariable analysis; work experience, work hours/day, previous injury status, managerial support on OSH, cigarette smoking, and job satisfaction were significantly associated with poor sleep quality.

Construction workers who had work experience of more than 2 years were 42% (adjusted odds ratio [AOR] = 0.58, 95% confidence interval [CI]: [0.34, 0.98], *p* = 0.042) less likely to experience poor sleep quality than workers who had ≤2 years work experience. It was also identified that participants with work hours greater than 8 h/day were three (AOR = 2.86, 95% CI: [1.78, 4.58], *p* = 0.00) times more likely to experience poor sleep quality than workers whose work hours were ≤8 h/day. Similarly, construction workers who had a history of occupational injury were three times (AOR = 3.28, 95% CI: [1.96, 5.49], *p* = 0.00) more likely to experience poor sleep quality than workers who did not have an occupational injury.

Moreover, participants who did not have managerial support on OSH were two times more likely to experience poor sleep quality compared to employees who had support from their managers (AOR = 2.08, 95% CI: [1.29, 3.33], *p* = 0.002). Individuals who smoked cigarettes were four times (AOR = 4.08, 95% CI: [1.17, 14.28], *p* = 0.028) more likely to have poor sleep quality compared to their counterparts. Finally, workers who did not have job satisfaction were about two times (AOR = 1.79, 95% CI: [1.11, 2.89], *p* = 0.017) more likely to have poor sleep quality than individuals who were satisfied with their jobs (Table 5).

**Table 5:** Factors significantly associated with poor sleep quality among building construction workers in Southern Ethiopia, 2020.

Variables	Sleep quality		Bivariable analysis		Multivariable analysis	
	Good (*n*)	Poor (*n*)	COR (95% CI)	*p*‐Value	AOR (95% CI)	*p*‐Value
Work experience (years)						
≤2	89	229	1.00		1.00	
>2	51	46	0.35 (0.22, 0.56)	0.000	0.58 (0.34, 0.98)	0.042
Work hours/day (h/day)						
≤8	85	81	1.00		1.00	
>8	55	194	3.70 (2.42, 5.67)	0.000	2.86 (1.78, 4.58)	0.000
Previous injury status						
Yes	28	150	4.8 (2.98, 7.74)	0.000	3.28 (1.96, 5.49)	0.000
No	112	125	1.00		1.00	
Managerial support on OSH						
Good	79	93	1.00		1.00	
Poor	61	182	2.53 (1.67, 3.85)	0.000	2.08 (1.29, 3.33)	0.002
Cigarette smoking						
Yes	4	29	4.01 (1.38, 11.64)	0.011	4.08 (1.17,14.28)	0.028
No	136	246	1.00		1.00	
Job satisfaction						
Good	88	107	1.00		1.00	
Poor	52	168	2.65 (1.75, 4.04)	0.000	1.79 (1.11, 2.89)	0.017

Abbreviations: AOR, adjusted odds ratio; CI, confidence interval; COR, crude odds ratio; OSH, occupational safety and health.

## 4 DISCUSSION

This is novel research to evaluate sleep quality and its related components among construction workers in Ethiopia using a validated PSQI tool. In this study, no participant reported using sleep medication in the previous month, and only one‐third (33.8%) reported having very bad subjective sleep quality. These findings are similar to other studies conducted in Northern Ethiopia.^19^ This could be due to cultural or behavioral factors that people did not seek treatment for this kind of illness (sleep disorder) and did not want to take medications for sleep‐related illnesses.

The PSQI global score revealed 66.3% of study participants struggle with poor sleep quality. This result lines up with research conducted in Jimma town among adults (65.4%)^20^ and among pregnant women in Northern Ethiopia (68.4%).^19^ On the other hand, it is higher compared to studies conducted among chronic illness patients in South Wollo, Ethiopia (36%),^21^ university students in Ethiopia (55.8%),^22^ and Myanmar's migrant workers in Malaysia (62.5%).^23^ Moreover, this finding is higher than the study conducted among construction workers in Southern India (33.9%).^24^ This disparity might be explained by differences in research setting and socioeconomic level of the groups. For instant, in the study conducted in South Wollo, the study setting was a hospital and the study population was chronic patients. The other reason for the discrepancy could be due to cultural and lifestyle differences among the populations. Evidence suggests that lifestyle preferences have a significant association with sleep quality, and this is proven in the Southern India study.^24^

On the contrary, this study finding is lower than studies conducted among prisoners in Mettu town, Ethiopia (77.1%),^25^ and among nurses in Northwest Ethiopia (75.5%).^26^ The found disparities between studies might be due to differences in living conditions and exposure to occupation hazards of the study population. Literature indicates that crowded living conditions could result in poor sleep quality than less crowded housing.^27^ The higher percentage of poor sleep quality among prisoners in Mettu town and nurses could be attributed to the above‐mentioned condition.

This study found that increasing work experience was associated with reduced odds of poor sleep quality. A construction worker who had more than 2 years of work experience was 42% less likely to encounter poor sleep quality than a worker who had ≤2 years of work experience. A similar association was reported in a study done in China.^28^ Longer work experience would make workers adapt to their work environment, resulting in low work‐related anxiety and fear, this would directly or indirectly improve their sleep quality.^29^

The current study also showed work hours as a significant factor in poor sleep quality. Workers with greater than 8 h of work hours/day were three times more likely to experience poor sleep quality compared to workers who work for ≤8 h/day. This result aligns with the research carried out in America among Latino farmworkers,^30^ and Myanmar migrant workers in Malaysia.^23^ Working long hours was linked to reduced sleep hours, trouble with falling asleep, and nonrestorative sleep. This phenomenon was corroborated and established by longitudinal research conducted on British civil servants.^31^ Furthermore, a higher duration of work might lead to accumulated fatigue that contributed to poor sleep quality.^32^

This study also indicated that prior history of work‐related injury at the construction site was another risk factor for poor sleep quality. Similar results were obtained from studies conducted in Shiraz, Iran^33^ and Basel, Switzerland.^13^ Physical and psychological traumas caused by prior work‐related injuries might affect sleep quality in negative ways. Additionally, workers who were injured might be on subsequent medications to alleviate pain, which might disturb their sleep patterns in consequence.

Construction workers who had poor managerial support on OSH were two times experiences poor sleep quality than workers who had support from their managers. The above result was similar to the study done in the United States.^34^ The reason might be that those who had less managerial support on OSH were linked to higher levels of anxiety and stress at work, and this leads them to poor sleep quality.^29^

In addition, this study showed that cigarette smokers had a high risk of poor sleep quality than nonsmokers. This was also strengthened by another study in Ethiopia.^35^ A similar result was also obtained from Jordan^36^ and China.^37^ The presence of nicotine substances in cigarettes disrupts sleep by raising heart rate and increasing alertness.^38^ Furthermore, a study done in China indicated cigarette smoking might impair cognition and this directly impacts sleep quality.^37^

Finally, this survey revealed that poor job satisfaction might increase the chance of experiencing poor sleep quality among workers. This finding is consistent with studies done in Iran^39^ and Turkey.^40^ The possible reason for this might be due to low job satisfaction among employees had a detrimental influence on their daily activities, resulting in worse job performance and poor sleep quality.

## 5 LIMITATIONS OF THE STUDY

This study might be prone to social desirability and recall bias. Moreover, it could be vulnerable to healthy worker bias due to the fact that workers with injury might not be at the construction sites during the study period. Furthermore, a causal relation could not be sure for some variables due to the nature of our study design, cross‐sectional.

## 6 CONCLUSION AND RECCOMENDATION

This study revealed that two‐thirds of construction workers had poor sleep quality. Shorter working years (≤2 years), longer working hours/day (>8 h), prior injury status, lack of managerial support on OSH, cigarette smoking, and lower job satisfaction were the risk factors for poor sleep quality among construction workers. Therefore, creating efficient health education and promotion by taking the identified associated factors into account might assist in reducing the burden of poor sleep quality among construction workers.

In addition, further studies are recommended to identify the predicting factors of poor sleep quality among construction workers using advanced study methodologies. Moreover, factors such as living conditions, psychological and other health‐related factors need to be assessed.

## AUTHOR CONTRIBUTIONS

**Debisa E. Wendimu**: Conceptualization; data curation; formal analysis; investigation; methodology; software; supervision; validation; writing—original draft; writing—review and editing. **Solomon G. Meshesha**: Conceptualization; formal analysis; supervision; visualization; writing—review and editing.

## CONFLICT OF INTEREST STATEMENT

The authors declare no conflict of interest.

## ETHICS STATEMENT

Ethical approval was obtained from the Institutional Review Board of Arba Minch University, College of Medicine and Health Sciences. Written informed consent was taken from each study participant. Confidentiality of information was kept properly.

## TRANSPARENCY STATEMENT

The lead author Debisa E. Wendimu affirms that this manuscript is an honest, accurate, and transparent account of the study being reported; that no important aspects of the study have been omitted; and that any discrepancies from the study as planned (and, if relevant, registered) have been explained.

## Supporting information

Supporting information.

## Data Availability

The data that support the findings of this study are available from the corresponding author upon reasonable request.

## References

[1] Colrain IM . Sleep and the brain. Neuropsychol Rev. 2011;21(1):1‐4. 21259122 10.1007/s11065-011-9156-z

[2] US Department of Health and Human Services . Your guide to healthy sleep. 2011 (p. 72).

[3] Watson NF , Badr MS , Belenky G , et al. Recommended amount of sleep for a healthy adult: a joint consensus statement of the American Academy of Sleep Medicine and Sleep Research Society. J Clin Sleep Med. 2015;11(6):591‐592. 25979105 10.5664/jcsm.4758 PMC4442216

[4] Ghods K , Abdoallahpour A , Ahmadi M , et al The relationship between sleep disorders and quality of life in rotating shift workers at a textile factory. Middle East J Rehabil Heal Stud. 2017;4(3):e12289.

[5] Kling RN , McLeod CB , Koehoorn M . Sleep problems and workplace injuries in Canada. Sleep. 2010;33(5):611‐618. 10.1093/sleep/33.5.611 20469803 PMC2864876

[6] Nishitani N , Kawasaki Y , Sakakibara H . Insomnia and depression: risk factors for development of depression in male Japanese workers during 2011–2013. Int J Public Health. 2018;63(1):49‐55. 29051985 10.1007/s00038-017-1043-9

[7] Stranges S , Tigbe W , Gómez‐Olivé FX , Thorogood M , Kandala NB . Sleep problems: an emerging global epidemic? Findings from the INDEPTH WHO‐SAGE study among more than 40,000 older adults from 8 countries across Africa and Asia. Sleep. 2012;35(8):1173‐1181. 22851813 10.5665/sleep.2012 PMC3397790

[8] Manzar MD , Bekele BB , Noohu MM , et al. Prevalence of poor sleep quality in the Ethiopian population: a systematic review and meta‐analysis. Sleep Breath. 2020;24:709‐716. 31183743 10.1007/s11325-019-01871-x

[9] Kim Y , Lee S , Lim J , et al. Factors associated with poor quality of sleep in construction workers: a secondary data analysis. Int J Env Res Public Health. 2021;18(5):2279. 33668985 10.3390/ijerph18052279 PMC7956805

[10] Lin Y , Liu S , Li S , Zuo H , Zhang B . Relationships between the changes in sleep patterns and sleep quality among Chinese people during the 2019 coronavirus disease outbreak. Sleep Med. 2022;91:154‐160. 33541805 10.1016/j.sleep.2021.01.021 PMC7813507

[11] Al‐Bouwarthan M , Quinn MM , Kriebel D , Wegman DH . Risk of kidney injury among construction workers exposed to heat stress: a longitudinal study from Saudi Arabia. Int J Environ Res Public Health. 2020;17(11):3775. 32466510 10.3390/ijerph17113775 PMC7312975

[12] Chung JWY , So HCF , Yan VCM , et al. A survey of work‐related pain prevalence among construction workers in Hong Kong: a case‐control study. Int J Environ Res Public Health. 2019;16(1404):1404. 31003525 10.3390/ijerph16081404 PMC6517896

[13] Uehli K , Miedinger D , Bingisser R , et al. Sleep quality and the risk of work injury: a Swiss case‐control study. J Sleep Res. 2014;23(5):545‐553. 24889190 10.1111/jsr.12146

[14] Altevogt BM , Colten HR , Institute of Medicine (US) Committee on Sleep Medicine and Research . Functional and economic impact of sleep loss and sleep‐related disorders. In: Colten HR , Altevogt BM , eds. Sleep Disorders and Sleep Deprivation: An Unmet Public Health Problem. National Academies Press; 2006:67‐72. 20669438

[15] Hafner M , Stepanek M , Taylor J , Troxel WM , Van Stolk C . Why sleep matters—the economic costs of insufficient sleep: a cross‐country comparative analysis. Rand Health Q. 2017;6(4):11. 10.7249/rhq-06-04-11 PMC5627640 28983434

[16] Buysse DJ , Reynolds CF , Monk TH , Berman SR , Kupfer DJ . The Pittsburgh Sleep Quality Index: a new instrument for psychiatric practice and research. Psychiatry Res. 1989;28(2):193‐213. 2748771 10.1016/0165-1781(89)90047-4

[17] Gebremeskel TG , Yimer T . Prevalence of occupational injury and associated factors among building construction workers in Dessie town, Northeast Ethiopia; 2018. BMC Res Notes. 2019;12(1):481. 31382990 10.1186/s13104-019-4436-4 PMC6683456

[18] Salahuddin M , Maru TT , Kumalo A , Pandi‐Perumal SR , Bahammam AS , Manzar MD . Validation of the Pittsburgh Sleep Quality Index in community dwelling Ethiopian adults. Health Qual Life Outcomes. 2017;15(1):58. 10.1186/s12955-017-0637-5 28347341 PMC5369003

[19] Jemere T , Getahun B , Tadele F , Kefale B , Walle G . Poor sleep quality and its associated factors among pregnant women in Northern Ethiopia, 2020: a cross sectional study. PLoS One. 2021;16(5):e0250985. 33945578 10.1371/journal.pone.0250985 PMC8096079

[20] Berhanu H , Mossie A , Tadesse S , Geleta D . Prevalence and associated factors of sleep quality among adults in Jimma Town, Southwest Ethiopia: a community‐based cross‐sectional study. Sleep Disord. 2018;2018:8342328. 29850261 10.1155/2018/8342328 PMC5937373

[21] Edmealem A , Ademe S , Andualem A . Sleep quality and associated factors among patients with chronic illness at South Wollo Zone Public Hospitals, Northeast Ethiopia. Clin J Nurs Care Pract. 2021;5:43‐50.

[22] Lemma S , Gelaye B , Berhane Y , Worku A , Williams MA . Sleep quality and its psychological correlates among university students in Ethiopia: a cross‐sectional study. BMC Psychiatry. 2012;12:237. 23270533 10.1186/1471-244X-12-237 PMC3554495

[23] Aye L , Arokiasamy J , Barua A , Yadav H , Onunkwor O . Prevalence and determinants of poor sleep quality among Myanmar migrant workers in Malaysia: a cross‐sectional study. Br J Med Med Res. 2017;19(12):1‐9.

[24] Sathvik S , Krishnaraj L , Awuzie BO . An assessment of prevalence of poor sleep quality among construction workers in Southern India. Built Environ Proj Asset Manag. 2023;13(2):290‐305. 10.1108/BEPAM-03-2022-0041

[25] Abdu Z , Hajure M . Prevalence and associated factors of poor quality of sleep among prisoners in Mettu Town prison, Oromia, South West Ethiopia, 2019. Open Pub Health J. 2020;13(1):94‐100.

[26] Segon T , Kerebih H , Gashawu F , Tesfaye B , Nakie G , Anbesaw T . Sleep quality and associated factors among nurses working at comprehensive specialized hospitals in Northwest, Ethiopia. Front Psychiatry. 2022;13:931588. 36051547 10.3389/fpsyt.2022.931588 PMC9425912

[27] Liu X , Liu L , Wang R . Bed sharing, sleep habits, and sleep problems among Chinese school‐aged children. Sleep. 2003;26(7):839‐844. 14655917 10.1093/sleep/26.7.839

[28] Zhao X , Zhang T , Li B , et al. Job‐related factors associated with changes in sleep quality among healthcare workers screening for 2019 novel coronavirus infection: a longitudinal study. Sleep Med. 2020;75:21‐26. 32853914 10.1016/j.sleep.2020.07.027 PMC7403128

[29] Molla A , Wondie T . Magnitude of poor sleep hygiene practice and associated factors among medical students in Ethiopia: a cross‐sectional study. Sleep Disord. 2021;2021:6611338. 33643669 10.1155/2021/6611338 PMC7902132

[30] Sandberg JC , Nguyen HT , Quandt SA , et al. Sleep quality among Latino farmworkers in North Carolina: examination of the job control‐demand‐support model. J Immigr Minor Health. 2016;18(3):532‐541. 26143366 10.1007/s10903-015-0248-3 PMC4710554

[31] Virtanen M , Ferrie JE , Gimeno D , et al. Long working hours and sleep disturbances: the Whitehall II prospective cohort study. Sleep. 2009;32(6):737‐745. 19544749 10.1093/sleep/32.6.737 PMC2690560

[32] Brossoit RM , Crain TL , Leslie JJ , Hammer LB , Truxillo DM , Bodner TE . The effects of sleep on workplace cognitive failure and safety. J Occup Health Psychol. 2019;24(411):411‐422. 30489101 10.1037/ocp0000139 PMC9036851

[33] Mani A , Dastgheib SA , Chanor A , Khalili H , Ahmadzadeh L , Ahmadi J . Sleep quality among patients with mild traumatic brain injury: A Cross‐Sectional study. Bulletin of emergency and trauma. 2015;3(3):93‐96. Available from https://www.ncbi.nlm.nih.gov/pubmed/27162910 27162910 PMC4771248

[34] Taouk L , Farrow VA , Schulkin J . Amount and quality of sleep: exploring the role of stress and work experience in a sample of obstetrician‐gynecologists. J Psychosom Obstet Gynecol. 2018;39(3):190‐195. 10.1080/0167482X.2017.1320985 28463031

[35] Birhanu TT , Hassen Salih M , Abate HK . Sleep quality and associated factors among diabetes mellitus patients in a follow‐up clinic at the University of Gondar comprehensive specialized hospital in Gondar, Northwest Ethiopia: a cross‐sectional study. Diabetes Metab Syndr Obes. 2020;13:4859‐4868. 33328747 10.2147/DMSO.S285080 PMC7734063

[36] Saadeh H , Saadeh M , Almobaideen W , et al Effect of COVID‐19 quarantine on the sleep quality and the depressive symptom levels of university students in Jordan during the Spring of 2020. Front Psychiatry. 2021;12:605676. 33664681 10.3389/fpsyt.2021.605676 PMC7920987

[37] Liu JT , Lee IH , Wang CH , Chen KC , Lee CI , Yang YK . Cigarette smoking might impair memory and sleep quality. J Formos Med Assoc. 2013;112(5):287‐290. 23660225 10.1016/j.jfma.2011.12.006

[38] AlRyalat SA , Kussad S , El Khatib O , et al. Assessing the effect of nicotine dose in cigarette smoking on sleep quality. Sleep Breath. 2021;25:1319‐1324. 33118055 10.1007/s11325-020-02238-3

[39] Hemmati‐Maslakpak M , Mollazadeh F , Jamshidi H . The predictive power of sleep quality by morning‐evening chronotypes, job satisfaction, and shift schedule in nurses: a cross‐sectional study. Iran J Nurs Midwifery Res. 2021;26(2):127‐132. 34036059 10.4103/ijnmr.IJNMR_301_19 PMC8132855

[40] Karagozoglu S , Bingöl N . Sleep quality and job satisfaction of Turkish nurses. Nurs Outlook. 2008;56(6):298‐307. 19041451 10.1016/j.outlook.2008.03.009

