# Supplementary material for: Factors associated with poor sleep quality among construction workers in Arba Minch town, Ethiopia: A cross‐sectional study
Source: Health Sci Rep. 2023 Nov 21;6(11):e1715. doi: 10.1002/hsr2.1715 (PMC10663431; doi:10.1002/hsr2.1715)
Supplement: Supplementary file 1 — Supporting information. [file HSR2-6-e1715-s001.docx]

**Appendix 1. Information sheet**

Good morning/ Afternoon.

My name is __________________________. Am working as data collector for the study being conducted in Arba Minch town on sleep quality and its associated factors among construction workers. I would like to ask you a few questions regarding your sleep quality status. The interview would take 15-30 minutes of your time. The purpose of this study is to assess your sleep quality and its associated factors among construction workers in Arba Minch town. Your participation in this study will contribute government and other concerned bodies for future planning and take appropriate intervention. Your participation is completely voluntary. You can refuse to answer any question and/or withdraw from the study at any time without a problem to you. All your responses will remain strictly confidential and your responses will not be linked to your identity at any time. I would like to inform you that there is no potential risk that can harm you and there is no any payment for participating in this study. If you have any question on what we have talked so far you welcome and if you have any question at any time you can contact the principal investigator through the following address:

Principal investigator: Debisa Eshatu

Mobile: 0913506056

E-mail: edebisa@gmail.com

Can we proceed? 1. Yes 2. No

**Appendix 2. Consent form**

I have read the participant information sheet. I have clearly understood the purpose of the research, the procedures, the risks and benefits, issues of confidentiality, the right of participation and the contact address for any queries. I have been given the opportunity to ask any questions for things that may have been unclear. I was informed that I can terminate the study at any time. Therefore, I declare my voluntary consent to permit this study to be conducted with my signature as indicated below.

Name: _____________________Date: _________________________

Signature of participant ___________________

Name: _____________________ Date: _________________________

Signature of the supervisor or principal investigator ______________

Thank you!

Note: This is to be signed face to face in the presence of the data collector.
